# Some Phases of Comparative Psychology

**Published:** 1888-01

**Authors:** Cornelius M. O’Leary


### EDITORIAL DEPARTMENT.

### SOME PHASES OF COMPARATIVE PSYCHOLOGY.

CORNELIUS M. O'LEARY, M. D., LL.D.

So profound and important are the changes which the methods of scientific investigation have undergone within the last quarter of a century, that they have imparted to the sciences themselves a new and distinct character. Theory and speculation have almost entirely given way to experience and observation, and hypotheses are framed only with a view to weaving facts into something like a logical and continuous whole. Scientific men have become exceedingly slow in arriving at conclusions, and many of them devote lives of assiduous toil to accumulating facts without venturing an opinion concerning their bearing on current scientific doctrines. Even Darwin, the father of the most significant theory to be found in the annals of philosophy, was very little prone to theorize, and expended his chief labor on the collation of scientific data. This reluctance to build up theories on slender basis, at once marks the appreciation of an extended and exhaustive observation, and the recognized necessity of withholding the judgment from inferences which a fuller supply of facts might not justify. Perhaps in no domain of science has this fact become more conspicuous than in the study of psychology, as it has been prosecuted among men and animals. The earlier methods of psychological enquiry being confined to the observation of subjective phenomena, as revealed in consciousness, gave forth halting and imperfect conclusions. The facts of memory, judgment, imagination and emotion were studied only in the mind of the enquirer, and, though subjected to the keenest analysis that the subtlest minds could employ, two difficulties of an almost insuperable character stood in the way of a steady and satisfactory progress. In the first place the object and subject of the science being necessarily identical in such a method, the mind of the investigator was divided between the effort to maintain the conscious condition which was to be examined, and the analysis to which that condition was subjected. Hence the results lacked the continuity and coherence, which are to be found in the physical sciences, and the tangible character of these was replaced by a subtle and baffling elusiveness. Moreover, subjective phenomena are not only variable in themselves, but differ in individual minds and assume protean shapes, as the moods of different observers are influenced by external and internal circumstances. There can be no question, therefore, that had the investigation of mental phenomena been limited to the observation of subjective facts, psychology would have remained an infant science and our knowledge of the mental condition of the lower animals would have been confined to curious anecdotes about dogs and horses and an occasional recurrence of the meaningless question, "Do the lower animals reason?" The first ray of light shed on modern psychology flashed with electric brightness from the physiological principle that every mental manifestation is accompanied by a change in some portion of the nervous structure and that such portion is organically related to the manifestation in question. From this principle speedily flowed the discoveries of Flourens, Bichat and Bell touching the origin of motion and sensation and their reference to the anterior and posterior roots as they emerge from the spinal cord. In this principle, likewise, we find the key to the localization of function and to the brilliant discoveries of Carpenter, Ferrier, Hitzig, Bastian and Luys. In fact, it at once led us to a knowledge of the organic union that subsists between the nervous system and mental states, and rendered possible a rational prosecution of human psychology from the earliest moments of fœtal existence through the period of adolescence up to that of maturity. It also opened the door to the labors of the comparative psychologist, and established an intelligent *rapport* between all psychical phenomena irrespective of the subjects in which they occur. It is no wonder that the psychology of the schools, the psychology to which Locke, Berkely, Malebranche, Hume, Mill, Reid, Stewart and Hamilton devoted the ceaseless labor of their lives was vaccillating, imperfect and unsatisfactory, when, owing to the rudimentary condition of physiology, these philosophers were debarred from the observation of human mental activity in its elementary states and as exhibited in animals of a lower order than man. Their study of mental facts was limited to the highly complicated operations of their own minds, or to the observation of similar phenomena in others. They were in the condition of one who should undertake the analysis of a watch or a steam engine by an observation of its functions without a disintegration of its component parts. The history of psychology is, consequently, the melancholy record of human endeavor put forth in its most energetic shape in the vain expectation of accomplishing the impossible. Aristotle, Plato and St. Thomas labored under difficulties which we, with good reason, deem amazing, and we cannot but admire the painstaking sagacity of those gifted men that enabled them to build up a system of mental science which, viewed from the standpoint of the subjective, is marvelous. Had they but understood the influence which nervous conditions exert upon mental manifestations, what a world of useless speculation might have been spared them, and how much more contributive to a subtantial scientific structure their labors would have proved. The inseparable connection, therefore, which exists between mental activity and the changes that occur in nerve cells and fibres being established, it is clear that a full and lucid understanding of the laws which govern these latter is indispensable to an adequate comprehension of mental function. Moreover, it is equally plain that human psychology can never reach its fullest development unless it proceeds *pari passu* with the facts which a study of psychical phenomena in the lower animals brings to light.

The co ordinate branches of science are found to be more closely interwoven and mutually dependent the more they are studied, and there is no fact for which we are more indebted to Comte and his positive philosophy, than the necessity which he has pointed out, of prosecuting our scientific inquiries in any direction with a reference to the influence they may have on cognate branches. Not only, therefore, do the facts of human fœtal existence possess a bearing on the complex phenomena of mature psychical life, but the contemplation of the lowest forms of living beings in which traces of nerve tissue are to be found, as in the Medusæ or jelly-fishes, also aids us in obtaining an adequate idea of the significance of mental activity. The necessity of this procedure, is illustrated by consideration of those mental acts which are common to all living beings, and which are connected with a condition of the nervous system to be found among all. Now, there is no fact in the range of mental phenomena so fundamental, or so general, as memory and its correlated form of associated ideas, and we consequently find throughout the entire animal kingdom a similar arrangement of ganglia and double fibres, together with innumerable connecting nervous arcs on the activity of which these functions depend. The older psychologists allowed to the lower animals a sort of memory which they called animal memory, but which they held to be essentially different from human memory. This untenable distinction is at once a proof of the erroneous idea these psychologists had of the essential nature of memory, and sets the seal of condemnation on those theories of memory which had passed current in the scientific world up to the time of Broussais. On its objective side we can perceive no difference between memory as exercised by man and as exhibited by animals of a lower order. In both instances an impression wrought upon a ganglion induces therein a permanent or organic change, not a mere phosphorescent picture that does not sink into the ultimate structure of the nervous substance, not a photographic impression which is merely on the surface, but a profound and vital change in the molecules of the cell, which so alters its character that its essential constitution is changed. When a similar impression is repeated the same condition no longer exists; the cells of the ganglion have become, as it were, orientated, and they respond to the repetition of the same stimulus with *some reference* to its first application. Not only does the stimulus traverse a route already exploited, but it does so all the more readily because the same stimulating current had already passed over it. In its simplest form this fact constitues the basis of organic memory, and because it is intimately connected with a vital process it has been appropriately designated by Rebot in his work on "Diseases of Memory," as biological. All secondary automatic functions, those in which the element of conscious attention is wanting, are the result of nervous discharges, frequently traversing the same nerve routes and by a harmonious activity bringing not only isolated ganglia and with their dependent passivities (muscles) into relation, but entire sets and systems of ganglia as well as nerve arcs and zones. The result of this highly complex operation is the establishment of corresponding automatic activities which make up the sum total of those daily actions which we perform unconsciously.

The ability to perform these actions is acquired in a manner precisely similar by men and beasts, and is an education of the muscular apparatus which gains in perfection and precision in proportion as the element of consciousness disappears. The muscles stand to the nerves as servants who discharge their various offices with all the greater ease and alacrity, according as they recognize with greater readiness the voice and authority of their master. Romanes and Rebot insist that true memory is exhibited in the performance of these secondary automatic actions, and that the absence of conscious attention merely enhances their memorative character. At this stage of the inquiry, much confusion results from the employment of terms which a spiritual psychology, if we may use the expression, has long since consecrated, and the effort to wrest these from their original signification has not been conspicuously happy. This is especially true in the case of those physiologists, who, like Maudsley, insist on basing a crude system of materialism on the recent discoveries that have been made in the domain of neurology. Whether consciousness is an added element of force proceeding from a source quite independent of the nervous system, or is but an expression of nervous activity in a nascent state, is a question that can have no influence on the normal changes that take place in the molecules of the nerve cells. The attempt to account for consciousness by any such changes is and must remain baffling. Nor is it necessary to propound the query in order that the complex and progressive changes in the nervous centers may be logically connected with memory. We know that the first discharges of nervous force that pass over a system of fibres, ganglia and connecting arcs, are made with difficulty and hesitancy, that the child who handles the pen or the bow for the first time, does so with much awkardness and painstaking, and that it is only when the route has been made smooth by repeated passages of nervous force, when the pioneer discharges have, as it were, overcome friction, levelled the hills and filled up the valleys, that facility and rapidity of movement are acquired. Now cons ious efforts are certainly required in order to overcome the difficulties that attend the first passages of nervous discharges over an unaccustomed route, but it by no means follows that the presence of consciousness as a factor in those rudimentary forms of memory detracts from their character or is the cause of their faltering, since it may, without trenching on probability, be regarded as a sentinel stationed on the road to guide the steps of the inexperienced wayfarer. When the traveler has become acquainted with the landmarks, and has located the sharp turns and steep inclines on the way, he needs no mentor at his side to make him heed.

But Maudsley and those who with him inveigh against the value of consciousness in memory, and claim that memory, in its higher manifestations, is independent of consciousness, mistake the character and functions of the latter and attack it because it does not possess attributes which psychologists of the spiritual school have never claimed for it. It is much more consistent with the true spirit of the scientific method, to avoid such metaphysical discussions in purely physiological researches, and when considering the objective side of mental phenomena, to keep steadily in view only the progressive changes that the nerve tissues exhibit in their development from the lowest forms of life to the complicated cerebral functions of the human subject. Certain it is that comparative psychology must await a still very remote period in the progress of its evolution before any of its stages can be elucidated by a discussion of the subtle and perplexing difficulties that surround the question of consciousness. The origin of consciousness can never be accounted for unless we admit the existence of a conscious substance, and that certainly is not the nervous tissue either in men or animals. We may connect conscious states with nerve change and determine the peculiar character of the change that is accompanied by special forms of consciousness, but the production of consciousness itself by which we recognize our own existence must ever remain a mystery among mysteries. Even Romanes, whose common sense is no less remarkable than his scientific acumen, loses himself in a quagmire of false reasoning while endeavoring to clear up this more than perplexing difficulty. He is right in stating that consciousness attends the performance of unaccustomed actions, that the nervous discharges which accompany such actions are compelled to traverse new and unbroken routes, and that nervous centres respond with less alacrity to their stimulation, but he evidently begs the question when he says that "the lengthening of the time of latency finds its psychological expression in the rise of consciousness."

Consciousness is not the result of the nervous function that is concerned in the production of a specific act, different from consciousness, and this is what Romanes would make of it. But, however we may view consciousness with respect to memory, the facts which attest the dependance of this faculty on permanent and organic alterations occurring in the nervous molecules are indisputable, and incidentally reveal the profound influence which the silent and incessant action of the brain exerts upon conscious life.

Education in man, training in the lower animals and the conservation of hereditary traits and instincts, are the partial expression of changes wrought in the nerve cells which are the unconscious condition of special states of consciousness. The far-sightedness and sagacity of the diplomat, the visions of the poet and the deep speculations of the philosopher, are not only intimately connected with molecular changes in the nervous structures, but each species of activity is represented by a special organic condition of nerve molecule with which that activity is associated. Instinct in like manner, which is reflex nervous action accompanied by consciousness depends for its characteristic manifestations on the special changes which the nerve cells undergo.

The innate ferocity of the kitten which, as Darwin observes, growls with hair erect at the first sight of a mouse and even trembles with excitement at the smell of one, is the result of stored up and transmitted nervous energy which has grown into definite shape and action in the course of many generations. These significant facts have received their true interpretation in the light which the study of physiological memory has shed upon them, and are but a fraction of the mental phenomena which admit of expl ination in the same manner. The lo aliza- tion of function is but another form of expression of the same fact, for it would be impossible to refer any function to a special nervous center, were we not aware that such a center had been thrown into a state of erethism on each occasion of functional activity, and had taken on in consequence a permanent and organic aptitude for a repetition of the performance.

In the study of comparative psychology, therefore, we cannot overestimate the important part which recent researches in the physiology of memory have played, and we are encouraged to hope that soon a larger and steadier light will be thrown by new discoveries on other mental processes which have hitherto been studied, only in the dim atmosphere of consciousness. But it is not alone in he modification of the nervous elements and the performance of the type they may have assumed that gives to memory its wonderful power of reproduction, but also the formation among those elements of determinate associations for each particular act, and the establishment of certain dynamic affinities which by repetition become as stable as the primitive anatomical connections. As Rebot aptly expresses it, nerve cells are like the letters of the alphabet, just as each one of which may enter into countless combinations and form a constituent part of numberless words, so the nerve cell may receive and retain numberless impressions by virtue of which it becomes part and parcel of innumerable memories. It is this harmonious play of activities amid an extensive and complicated apparatus, this interchange of energy among correlated bands of sympathetic nerve cells, that gives to the study of the objective side of memory its interest and importance, and establishes its character as a true biological process.

By the aid of the knowledge which mental physiology supplies we become acquainted with the conditions on which the healthy exercise of memory depends, we recognize the character and tendencies of its aberrations and are enabled to take measures against the destructive processes that threaten it. By the same light also we are enabled to analyze the complicated processes of conscious memory, resolve it into component elements and form an appreciation of the relative importance of each and every factor in the function. Indeed, this ability to simplify and divide the lines of inquiry in comparative psychology, is one of the chief advantages which the physiological method has bestowed upon the science, and has already wonderfully widened its domain. The most complicated of our mental actions is simple on its subjective side, or, so far as consciousness reveals to us its character, while on its physiological side, it involves a complexity of processes and an intricate series of adjustive movements most difficult to follow and understand. In order to study these to advantage it is necessary to consider them separately and to note each successive mental step in the psychological whole that follows. The mind will thus be enabled to connect casually subjective phenomena that stand apparently unrelated, and to view them as elementary components of a mental process which consciousness alone proclaims to be simple and indivisible. Throughout these investigations all speculation touching the ultimate causes of mental action should be avoided, unless fully warranted by the facts, and thus the psychologist of the old school may conduct his lines of inquiry by the aid of a new and welcome light, while the physiologist may win the renown of invaluable discoveries and contributions to psychology without needlessly attacking the doctrine of a spiritual soul or substance.

